# Element Accumulation Patterns of Native Plant Species under the Natural Geochemical Stress

**DOI:** 10.3390/plants10010033

**Published:** 2020-12-25

**Authors:** Vladimir A. Alekseenko, Natalya V. Shvydkaya, Alexey V. Alekseenko, Maria M. Machevariani, Jaume Bech, Mariya A. Pashkevich, Alexander V. Puzanov, Aleksey V. Nastavkin, Núria Roca

**Affiliations:** 1 Institute of Earth Sciences, Southern Federal University, 344006 Rostov-on-Don, Russia; vl.al.alekseenko@gmail.com (V.A.A.); geo_alex@mail.ru (A.V.N.); 2 Department of Botany and General Ecology, Kuban State Agrarian University, Krasnodar, 350004 Krasnodar Krai, Russia; nepeta@mail.ru; 3 Department of Geoecology, Saint Petersburg Mining University, 199106 Saint Petersburg, Russia; mpash1963@yandex.ru; 4 Department of Mineralogy, Crystallography, and Petrography, Saint Petersburg Mining University, 199106 Saint Petersburg, Russia; wmdmaria@gmail.com; 5 Faculty of Biology, University of Barcelona, 08002 Barcelona, Spain; jaumebechborras@gmail.com (J.B.); nroca@ub.edu (N.R.); 6 Institute for Water and Environmental Problems SB RAS, 656038 Barnaul, Russia; puzanov@iwep.ru

**Keywords:** metal anomalies, trace elements, metal uptake, vegetation stress, hyperaccumulation, phytoremediation

## Abstract

A biogeochemical study of more than 20,000 soil and plant samples from the North Caucasus, Dzungarian Alatau, Kazakh Uplands, and Karatau Mountains revealed features of the chemical element uptake by the local flora. Adaptation of ore prospecting techniques alongside environmental approaches allowed the detection of geochemical changes in ecosystems, and the lessons learned can be embraced for soil phytoremediation. The data on the influence of phytogeochemical stress on the accumulation of more than 20 chemical elements by plants are considered in geochemical provinces, secondary fields of deposits, halos surrounding ore and nonmetallic deposits, zones of regional faults and schist formation, and over lithological contact lines of chemically contrasting rocks overlain by 5–20 m thick soils and unconsolidated cover. We have corroborated the postulate that the element accumulation patterns of native plants under the natural geochemical stress depend not only on the element content in soils and the characteristics of a particular species but also on the values of ionic radii and valences; with an increase in the energy coefficients of a chemical element, its plant accumulation decreases sharply. The contribution of internal factors to element uptake from solutions gives the way to soil phytoremediation over vast contaminated areas. The use of hyperaccumulating species for mining site soil treatment depends on several external factors that can strengthen or weaken the stressful situation, viz., the amount of bedrock exposure and thickness of unconsolidated rocks over ores, the chemical composition of ores and primary halos in ore-containing strata, the landscape and geochemical features of sites, and chemical element migration patterns in the supergene zone.

## 1. Introduction

### 1.1. Geochemical Stress

Since the middle of the past century, the term stress has been used quite often in medical and later environmental literature [1]. The response of various biological systems to the impact of extreme external environmental factors-stressors-is usually assumed in such cases. Geochemical stress occurs when external factors result in abnormal changes in the content of chemical elements or compounds, often accompanied by changing distribution and modes of occurrence. In most cases, such natural and technogenic transformations that affect biological and biomineral systems arise in the atmosphere, at the surface and in groundwater, and soil [2,3].

The issue of the vegetation response to geochemical fluctuations was initially discussed in relation to mineral exploration and detection of anomalies, with the thought-provoking works coupling geochemical stress studies and remote sensing [4,5,6,7,8]. Together with the geological search for mineralized sites, environmental concepts have been developed as well; current findings point to using spectrometric studies of the vegetation index as a tool to detect metal stressed plants [9,10,11].

Field surveys linked geochemical stress to trace element background values [12,13] and pollution levels [14]. During their evolutionary development, many organisms have adapted to the influence of a number of factors and can develop relatively normally under changing conditions [15]. In such cases, they are tolerant to stress; i.e., they can resist various extreme factors affecting them. Recent studies show transformations of flora and fauna under geochemical stress situations even in retrospect [16,17,18]. Furthermore, one of the most promising geochemical stress research areas is the biogeochemical consideration of hyperaccumulating species in view of ecofriendly soil remediation [19,20,21,22]. These studies show that the greatest changes in the content of elements in organisms under the influence of natural factors occur proximal to weathered deposits and with a significant increase in the concentrations of several elements in the growing environment. With the highest concentration of metals in ores over large areas, up to 90% of the plants cannot adapt to such conditions and disappear, leaving bare soil, such as in areas of boron and pyrite ores discussed by Ginzburg [23]. Some plants, on the contrary, need the high concentrations of certain elements in the growing environment for normal development [24,25,26,27].

### 1.2. Geochemical Provinces and Fields

The term “geochemical province” coined by A.E. Fersman [28] is one of the geochemical zoning units of the heterogeneous Earth′s surface: an area of certain associations of chemical elements that are in high or low concentrations in rocks, and, accordingly, in soils, and groundwater. As a result, geochemical conditions for the vegetation cover of each province differ from those of neighboring territories, meaning plants are under stress. A response to such situations, even by stress-tolerant plants, is the increased or reduced accumulation of chemical elements, either by the entire plant or by certain parts of it. In general, changes in the geochemical conditions of plant growth contribute to the formation of geobotanical or biogeochemical areas with different content of certain chemical elements in plants. To assess the changes, we conducted studies covering the North Caucasus geochemical province, as well as the Dzungarian Alatau Mountains, the Kazakh Uplands, and the Karatau Mountains. 

Closely located and genetically linked different-sized deposits are considered as mineral areas and nodes. The landscapes adjoining the deposits are characterized by certain geochemical patterns. First, many chemical elements in high concentrations (direct and indirect indicators of mineralization) are usually extremely unevenly distributed within their boundaries. The elements make up ore occurrences, areas of rudimentary ore formation, primary halos, and points of scattered mineralization. Secondly, leaching zones with significantly reduced content of indicator elements are found in rocks within the areas and nodes of deposits. 

This usually contributes to an uneven distribution of elements within the primary geochemical fields of concentration and redistribution of elements, corresponding to areas and nodes of mineral deposits [29]. Weathering of rocks of primary geochemical fields contributes to the formation of secondary pedochemical and biogeochemical fields. These fields can be geochemically stressful for native plant species, which results in the transformation of element accumulation patterns.

## 2. Materials and Methods

### 2.1. Study Purpose and Objectives

The biogeochemical studies aimed at revealing features of the chemical element uptake by the local flora. Thus, we formulated the research question as follows: when native plants are affected by the natural geochemical stress, what factors determine their element accumulation patterns, except for the element content in soils and the characteristics of a particular species?

The research objectives included the valuation of (i) the roles of element properties such as ionic radii, valences, and energy coefficients; (ii) several external factors that can strengthen or weaken the stressful situation, like the amount of bedrock exposure and thickness of unconsolidated rocks over ores, the chemical composition of ores and primary halos in ore-containing strata, the landscape and geochemical features of sites, and the chemical element migration patterns in the supergene zone.

### 2.2. Study Areas and Sampling Procedures

The landscape–geochemical exploration covered four major geographic areas: the North Caucasus, Kazakh Uplands, Dzungarian Alatau, and Karatau Mountains. The investigation was conducted on a scale from 1:500,000 to 1:10,000 and included the sampling of plants, topsoil horizons, and bedrocks, with a total of 20,038 specimens. The study incorporated the data gathered from the analysis of leaves and one-year needles; when examining the grass cover, shoots were taken. The major part of the anomalies identified as a result of the search for mineral deposits, including those identified by biogeochemical methods, was checked by mining or at least drilling operations. This allowed the discovery of numerous deposits and promising ore occurrences. The authors also carried out annual biogeochemical and lithogeochemical environmental assessment over 15 years in the areas of unreclaimed abandoned ore and nonmetal mines. In these cases, the sampling step varied from 2–5 to 200 m.

The North Caucasus geochemical province consists of two geobotanical areas [30], which have been studied to understand the floral element accumulation patterns. (i) The Caucasian area includes grasslands—fescue (*Festuca*), reed grass (*Calamagrostis*), bentgrass (*Agrostis*), etc. and broadleaved forests—oak (*Quercus*), hornbeam (*Carpinus*), beech (*Fagus*), chestnut (*Castanea*), ash (*Fraxinus*), fir (*Abies*), and pine (*Pinus*). Much less common mountain steppes are formed by fescue, feather grass (*Stipa*), and several types of shrub vegetation. (ii) The extremely diverse vegetation cover of the Black Sea area includes communities of the Mediterranean-type, including oak and juniper–pistachio woodlands (downy oak, *Quercus pubescens* Willd.), relict pines (*Pinus pityusa* Steven, *P. pallasiana* D. Don), upland—xerophytic steppes (fescue and feather grass), oak, beech, and chestnut forests, littoral communities (wild rye, *Leymus racemosus* ssp. *sabulosus* (M. Bieb.) Tzvelev), and high-mountain vegetation of subalpine, alpine, and subnival zones.

The Dzungarian Alatau is a region of foothills and ridges with the vegetation cover represented by post-forest sedge-wormwood-fescue steppes (*Festuca valesiaca* Gaudin, *Artemisia sublessingiana* Krasch. ex Poljakov, *A.* frigida Willd., and *Carex dimorphotheca* Stschegl.) complemented by meadowsweet-barberry-honeysuckle shrubs (*Spiraea hypericifolia* L., *Berberis sphaerocarpa* Kar. et Kir., and *Lonicera microphylla* Willd. ex Schult.). The landscapes are used as pasture, often subjected to fires and other anthropogenic impacts, and therefore, weed species like sophora, virgate wormwood, and viper’s bugloss are common (*Pseudosophora alopecuroides* (L.) Sweet, *Artemisia scoparia* Waldst. et Kit., and *Echium vulgare* L.).

The Kazakh Uplands include intermountain depressions covered by fescue-feather grass steppes with depleted xerophytic forbs such as sage and galatella (*Stipa sareptana* A.K. Becker, *S. lessingiana* Trin. et Rupr., *Salvia deserta* Schangin, *Galatella villosa* (L.) Rchb. f.) and sandy gravel slopes partially covered by shrubby steppes with meadowsweet and pea tree (*Spiraea hypericifolia* L., *Caragana frutex* (L.) K. Koch). 

The Karatau Mountains comprise a semidesert plateau cut by narrow V-shaped creek valleys. Savannoids developed in the area are an original vegetation type of high landscape significance. Plant communities are composed of tree-shrub species such as acer, apple, hawthorn, juniper, cherry, dog rose, and meadowsweet (*Acer*, *Malus*, *Crataegus*, *Juniperus*, *Cerasus*, *Rosa*, and *Spiraea hypericifolia* L.), suffruticulose wormwood inclusions (*Artemisia terrae-albae* Krasch. and *Artemisia sublessingiana* Krasch. ex Poljakov), and tall herbaceous vegetation like beardgrass, wheatgrass, inula, ferula, and prangos (*Bothriochloa ischaemum* (L.) Keng, *Elytrigia trichophora* (Link) Nevski, *Inula macrophylla* Kar. et Kir., *Ferula*, and *Prangos*). Fescue-feather grass steppes with sophora and saltwort form large massifs (*Festuca valesiaca* Gaudin, *Stipa capillata* L., *S. lessingiana* Trin. et Rupr., *Pseudosophora alopecuroides* (L.) Sweet, and *Salsola arbuscula* Pall.).

### 2.3. Data Acquisition and Interpretation

The chemical composition of 12,724 soil samples, 6289 samples of leaves and needles, and 1025 bedrock samples was determined by spectral emission analysis in a certified and accredited laboratory of Rosgeologia and the Common Use Centre of the Saint Petersburg Mining University. The applied analytical techniques followed the recommendations and experience of the previous studies in the field [31,32]. To distinguish anomalies in plants on the same principle as anomalies in soils, we obtained the data by the analysis of 729 averaged samples using a method with a double experiment repetition. For a part of the samples, X-ray fluorescence and neutron activation analyses were additionally performed. Intralaboratory and external control each accounted for 3–5% of the number of ordinary samples. Laboratories of the Institute of Biosphere Geochemistry (Novorossiysk), Magadangeologia (Magadan), the Institute of Geology of Ore Deposits, Petrography, Mineralogy and Biochemistry RAS (Moscow), and the Institute of Physical and Organic Chemistry and Department of Soil Science and Land Assessment of the Southern Federal University (Rostov-on-Don) performed the external control. This research was carried out as a part of the program of supporting the publication activity of the Southern Federal University. The calculation of analytical errors allowed us to consider the work of laboratories as good and less often satisfactory. In addition to our own figures, the published works of numerous researchers were widely applied to interpret and discuss the data obtained [33,34,35]. 

Following Vernadsky [36], we attribute the average content of chemical elements characterizing geochemical provinces to regional abundances, and the ratio of the measured concentration to the respective global or regional abundance as the enrichment factor (EF) and the depletion factor (DF) if calculated as a multiplicative inverse. This coefficient has been revised and still applied as a geochemical criterion [37]. The biological absorption coefficient (BAC), first used by Polynov [38], is another indicator of the response of plants to stressful situations in the growth environment. BAC is the ratio of the chemical element content in plant ash to its content in soils. The Cartledge ionic potential is the ratio of charge to radius, *Z/R_ion_* [39,40]. When potential values are less than three, complex ions are not formed, and cations easily pass into natural aqueous solutions; i.e., they become the most accessible for organisms. For values from three to 12, complex ions and slightly soluble hydrolyzed compounds are formed. If the Cartledge ionic potential is greater than 12, complex oxygen-containing, highly soluble anions are formed. For anions, energy coefficients are calculated based on an element’s valence V and its ionic radii R as (1):

Energy Coefficient = V^2^/2 × R_ion_, (1)

and following the Formula (2) for cations:

Energy Coefficient = V^2^ × (0.75 × R_ion_ + 0.2)/2 × R_ion_ (2)

where the ionic radii are given in angstroms [28]. Ions with high coefficient values precipitate earlier from solutions and, as they are less mobile, more often remain accumulated in eluvium.

## 3. Results and Discussion

### 3.1. Regional Abundances in Soils and Plants

The natural geochemical stress has the most intensive and long-term effect on plants through the elevated and lowered concentrations of chemical element associations in the soils of certain geochemical provinces in comparison with the Earth′s abundances. Let us consider the results of such an impact on the example of the North Caucasus geochemical province. For it, the ranked EF series of the chemical elements in soils is Pb_3.7_–Cu_2.6_–Zn_2.4_–Co_2.2_–Li_1.8_–Sc, Mn, Mo_1.4_–Ba, Ni, Ti_1.2_–V_1.1_; the DF series in soils is Ag_3.9_–Y_2.5_–Ge_2.3_–Cr_1.8_–Sn_1.7_–Zn, Ga_1.6_–Be_1.5_. Elements with the largest regional abundances—Pb, Cu, and Zn—are cationic aquatic migrants, highly and moderately mobile in an oxidative environment. Judging by the Cartledge ionic potentials [39,40], they migrate predominantly as complex ions; rather high values of ionic radii and relatively low energy coefficients allow migration over long distances. For Cu and Zn, biogenic accumulation plays a significant role. Thus, internal factors contribute to the plant accumulation of these elements over vast areas. 

However, EFs and DFs of the elements in the plants of the province do not coincide with the respective EFs and DFs calculated for the soils. The ranked EF series for the plant ash in the province differs significantly from the series for soils: Sr_12.0_–Sc_10.0_–Ba_8.2_–Nb_7.6_–Zr_7.0_–Yb_3.0_–Y_2.1_–Li_1.5_; the same differences were found for the DF series: Ge_50.0_–Ga_38.4_–Mo_15.4_–Zn_7.1_–Cr_5.2_–V_5.0_–Co_4.3_–Cu, Be_3.0_–Sn_2.5_–Mn_2.0_. The concentrations of soil-accumulated elements such as Cu, Zn, Co, Mn, Mo, and Ni are lower in plants of the province than the respective global abundances. However, Cu and Zn may be accumulated in plants for physiological reasons [29].

With more than 4000 samples analyzed, we believe that this can be explained by the following reasons: (i) a combined effect of several abnormal chemical element contents (or element association) in the soil on the accumulation of each of them by plants; (ii) changing modes of occurrence of elements in soils over various rocks; and (iii) typical ratios between element concentrations in plants. Even a relatively small quantitative change in the external conditions of plant growth, but developed over a large area, associated with the characteristics of geochemical provinces, causes a fairly significant change in the content of elements in plants. For example, in the soils of the North Caucasus province, the content of Sc exceeds its global abundance by 1.4 times, and in the plant ash the regional levels are 10 times higher than the global abundance in plants of the Earth [41,42].

### 3.2. Element Uptake in the Areas of Lithogeochemical Anomalies

**Figure 1:**
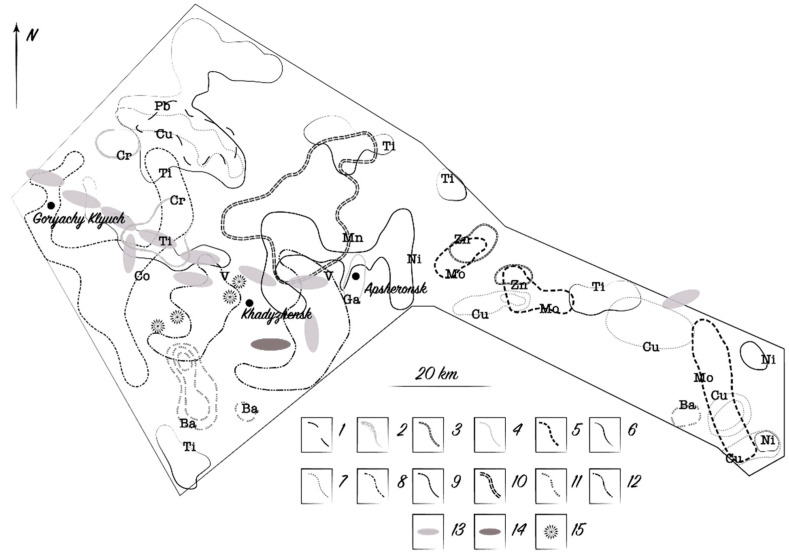
Secondary pedochemical metal anomalies over the partially overlapping mercury, oil, and gas fields. Symbols: 1—Pb; 2—Cr; 3—Zn; 4—Ga; 5—Mo; 6—Ti; 7—Cu; 8—Co; 9—Ni; 10—Mn; 11—Ba; 12—V; 13—gas field; 14—oil field; 15—mercury deposit.

A geochemical field is a smaller area than a geochemical province, and most commonly corresponds to areas of mineralization. The average content of indicator elements in rocks, soils, and plants of such fields is higher than in other parts of provinces. However, within each field, areas with normal, low, and high contents are distributed unevenly and the fields represent a number of close and partially overlapping anomalies, which is seen in Figure 1. Almost all the anomalies are low-contrast and are detected mainly by a large number of samples with concentrations equal to (3) and (4):

(3) $$C_{a}=\bar{x}\pm \frac{3S}{\sqrt{m}}$$

and

(4) Ca=ant(lg¯x¯)ε3m.

The lower limit of abnormal contents is the value calculated for nine correlating samples (*m* = 9) taken within the province. Thus, the content of indicator elements in the 30 cm topsoil horizon is only slightly higher than the regional abundance in the province’s soils. 

**Figure 2:**
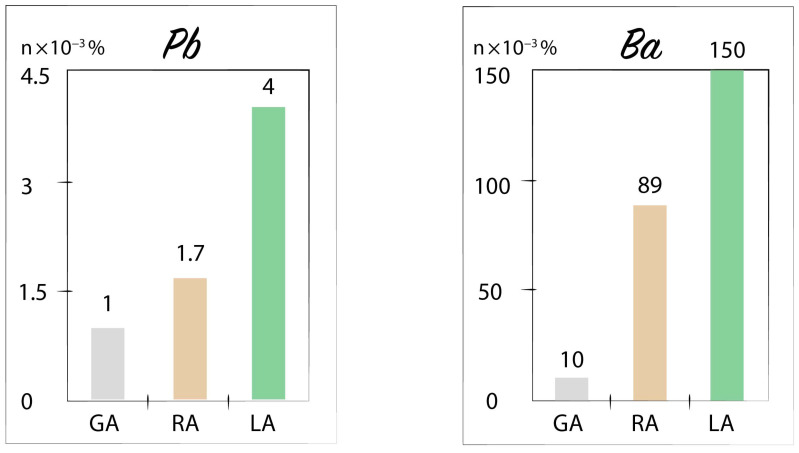
Metal accumulation in different milieu: GA—global abundance in the plants of Earth (after Kabata-Pendias; Tkalich [41,42]); RA—regional abundance of the North Caucasus geochemical province; LA—local average content in the ash of hornbeam (*Carpinus*) growing over mercury, oil, and gas fields.

We distinguished anomalies in plants on the same principle as anomalies in soils. They are also usually slightly higher than the regional abundance. For instance, in the ash of hornbeam leaves, the Pb content reaches 3–5 × 10^−3^%, while the global abundance for plants is 1 × 10^−3^% and the regional abundance of the province for hornbeam is 1.7 × 10^−3^%. For Ba, these values are 150 × 10^−3^%, 10 × 10^−3^%, and 89 × 10^−3^%, respectively (Figure 2). The location of anomalies of the same metal in the soil and the ash of leaves, as a rule, does not completely coincide spatially, even though many anomalies partially overlap each other. In the same field, the number of chemical elements that form lithogeochemical anomalies is marginally larger than their number in biogeochemical anomalies.

Indicator elements that form litho- and biogeochemical fields in the same area may differ significantly. The secondary lithogeochemical field in the southwestern part of the North Caucasus province with the known Pb, Zn, and Cu mineralization is represented by Ba, Cu, Mn, Ni, Ti, V, Cr, Ga, Mo, and Zn [43]. At the same site, the ash of hornbeam leaves shows no anomalous concentrations of indicator elements such as Cr, Ga, Mo, or Zn, but there are anomalies for Sr, Pb, and Zr that are absent in soils. The general data on the features of the accumulation of chemical elements by plants in the geological and geochemical situation of ore fields are given in Table 1. 

The ranked EF values for the ash of woody plants (which make up more than 90% of the total mass of all plants in the region) in various areas of mineralization in the North Caucasus are shown in Table 1. Even though the major ore deposits differ in geochemical features and genesis, it is possible to identify common chemical elements with the highest concentration in plants growing in geochemical fields compared to the whole province. We believe that Mo, Cu, Zn, Sr, Co, and W can be considered as such elements. Their EFs vary within the following limits: Mo—from traces to 3.8; Cu—from 1.6 to 2.2; Zn—from 1.8 to 2.8; Sr—from 1.4 to 2.2; and Co—from 1.4 to 2.3. Sr is the only element in the row that is accumulated in the province′s plants outside the fields. Therefore, the levels of these elements are not inherited from the geochemical patterns that characterize the province. For all these elements (except for W), biogenic accumulation plays a significant role [44] and owing to the ionic radii and energy coefficients, they can migrate at a long range in solutions. With this, we believe that plants could obtain and accumulate the listed elements in the most accessible form from relatively distant deposits, ore occurrences, and mineralization zones. Judging by the BACs values greater than unity (Table 2), woody vegetation of these geochemical fields is a barrier to Sr, Mn, Cu, Zn, and Ag, regardless of the type of geochemical field.

### 3.3. Plants of Explored and Mined Deposits

Mineral deposits are generally the most important natural geological and geochemical stressors. However, in the vast majority of cases, data on the response of biogeochemical reaction of plants are obtained after geological exploration, or after mining has commenced [45,46,47,48]. In this section, we discuss information about the overall reaction of plants to the deposits themselves, and man-made changes associated with exploration and mining [49,50,51,52,53,54,55,56]. In this regard, this section of the work deals with the biogeochemical features of plants tested before and after exploration (certain fields were discovered with our participation during the search by biogeochemical methods).

Biogeochemical studies of the Kazakhstani Pb-Zn deposits (the Telmanskoe (the Dzungarian Alatau), Bugunskoe (the Karatau Mountains), and Alkamergenskoe (the Kazakh Uplands)) revealed that the contents of the indicator elements above mineralization depend on a number of external and internal factors that can strengthen or weaken the stressful situation. 

Where deposits are unevenly exposed, such as in mountainous regions, the metal content of plant ash varies widely, from minimally abnormal for nine correlating samples to *n* × 0.01%, and rarely as much as *n* × 0.1%. In areas of contiguous unconsolidated cover, the element concentrations in plants do not fluctuate and depend on the chemical (Table 3) and mineral (Table 4) composition of ores. High-grade and low-grade ores normally affect the biogeochemical response of plants if the thickness of unconsolidated cover does not exceed 3 m. It is at this thickness of overlapping strata that the content of indicator elements in plants is also influenced by the ore composition (Table 4). The content of indicator elements in plants in stressful situations is also significantly influenced by the plant species [57]. For example, the Pb content can change more than three times for plants over high-grade zones (Table 3).

**Figure 3:**
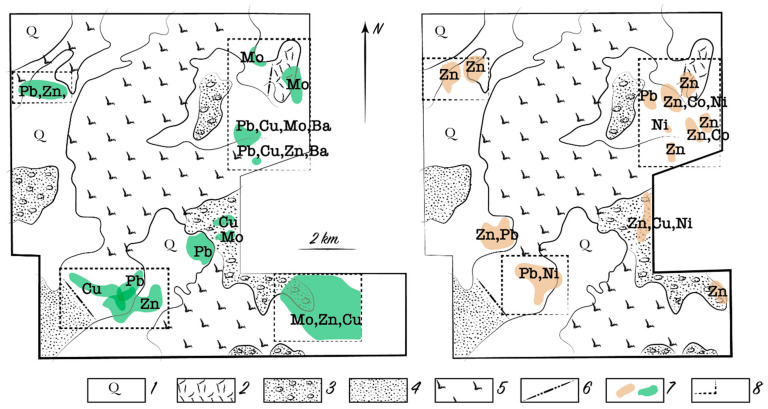
Biogeochemical (**left**) and deep lithogeochemical (**right**) anomalies corresponding to the Pb-Zn deposit below the 20 m thick loose deposits. Symbols: 1—recent loamy and sandy loamy sediments; 2—Lower and Middle Devonian effusive felsic rocks; 3—Paleogene quartzitic sandstones, sands, and pebbles; 4—Llandovery red-bed sandstones and gritstones; 5—Terreneuvian and Miaolingian effusive mafic igneous–sedimentary rocks; 6—disjunctive faults; 7—anomalies; 8—outlines of anomalous sites.

The thickness of the loose cover, especially superimposed, affects the degree of plant stress. Biogeochemical prospecting at the Alkamergenskoe ore field of the Kazakh Uplands identified a promising ore occurrence in an area with an average thickness of loose cover of 20 m. The presence of mineralization was confirmed by subsequent drilling of wells on a grid of 40 × 200 m^2^ (Figure 3). The content of Pb and Zn in the bedrock was 1–5%; however, no anomalies were detected in the upper soil horizons. The Pb content in the ash of feather grass (*Stipa*) over ore zones ranged from 0.01 to 0.023%, Zn from 0.02 to 0.04%, and Mo from 0.001 to 0.003%. We believe that the average and anomalous contents presented in Table 5 depend on the landscape–geochemical conditions over the field.

**Figure 4:**
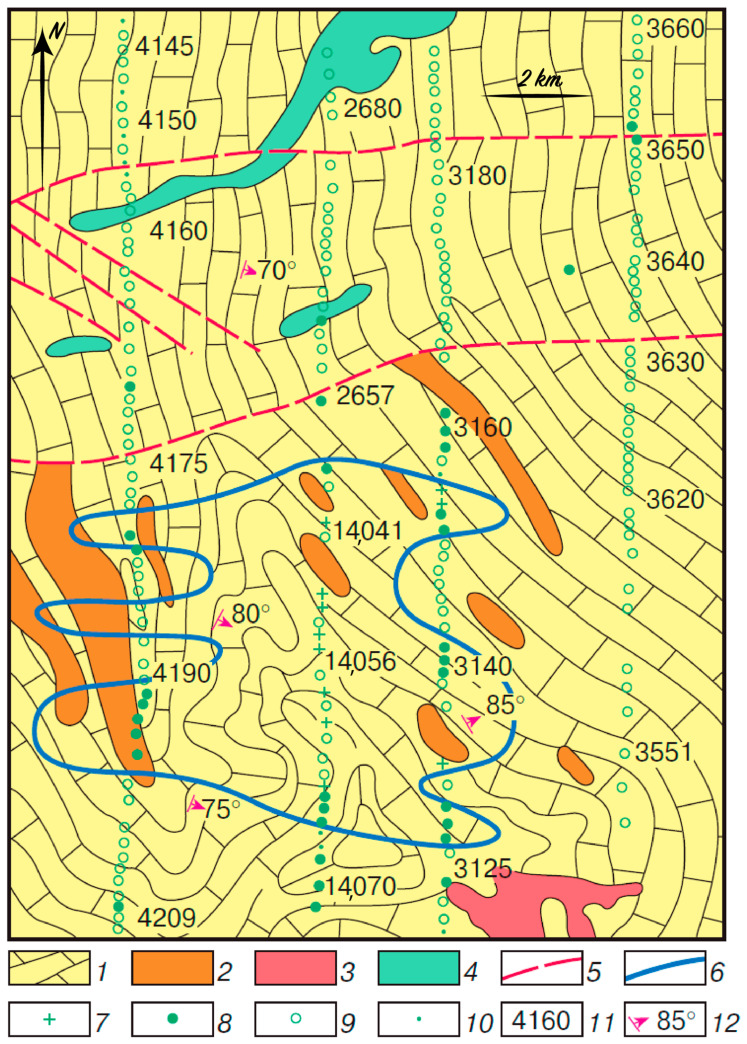
“Negative” biogeochemical Mo anomaly over the Telmanskoe Pb–Zn deposit. Symbols: 1—limestones; 2—ores and ore-hosting dolomites; 3—granites; 4—diabase porphyrites; 5—disjunctive faults; 6—outlines of anomalies; 7–10—sampling sites and Mo content (%) (7, below 5 × 10^−4^; 8, 5 × 10^−4^; 9, *n* × 10^−3^; 10, *n* × 10^−2^); 11—sample numbers; 12—dip and strike of rock layers.

In some instances, a geochemical stress in plants over ore deposits leads to an increased element uptake by plants, as well as causing a significant decrease or increase in the content of other elements due to the biological relationship between elements in organisms. The relationship between Pb and Mo is an example of this phenomenon. Somewhat simplistically, it can be explained by the purely biological functions of these elements. Molybdenum is a part of a large group of cell energy exchange enzymes, and Pb inhibits the formation of these enzymes. With relatively low absorption of Pb in a plant, the Mo content increases. A positive correlation of the contents of these metals in plants is an evidence of the normal viability of the organism that responds to toxic concentrations of Pb by enhanced formation of enzymes. Excessive Pb uptake (various species have different thresholds) leads to a decrease in the amount of Mo required by the plant. When this happens, a negative correlation exists between Pb and Mo. This process leads to “negative” biogeochemical Mo anomalies in plants over complex polymetallic deposits (Figure 4) and areas with technogenic Pb contamination. It should be noted that the Mo content did not decrease in the ores, surrounding formations, and in the contamination zones [58].

**Figure 5:**
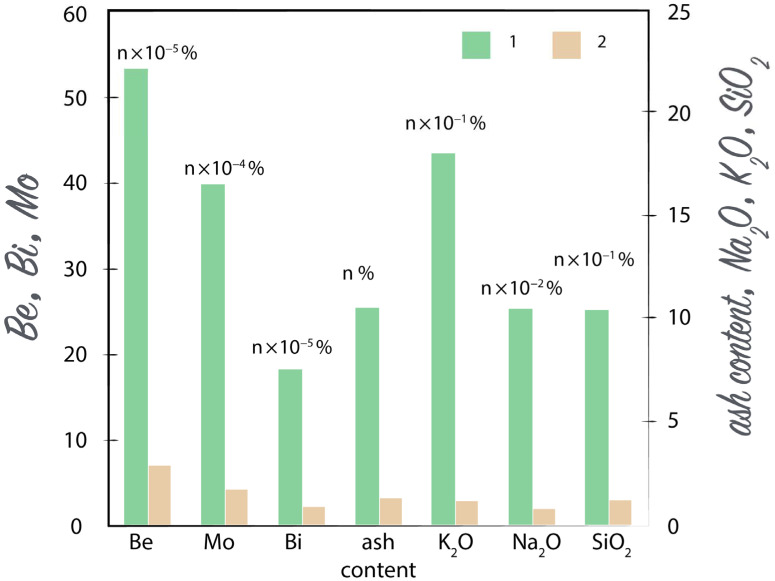
Distribution of Be, Mo, Bi, ash, K_2_O, Na_2_O, and SiO_2_ content in the ash of meadowsweet (*Spiraea hypericifolia* L.) over the ore field (1) and beyond the ore field (2).

Another biological relationship is the element ratios in plants, such as wormwood and meadowsweet of the rare metal deposit in the Kazakh Uplands. An increase in the concentration of Be, Mo, and Bi in soils and then in plants (by *n ×* 10^−3^%) leads to a sharp increase (*n ×* 10^−2^%–*n ×* 10^−1^%) of the listed oxides in the plant ash, and, accordingly, to an increase in ash content (Figure 5). At the same time, the content of K, Na, and Si in the soil medium and their modes of occurrence remained the same.

After the geological exploration, the chemical elements that make up the ore and the primary halos enter the supergene zone, where they are at much higher concentrations and of wider extent. This creates a special stressful geochemical situation. Table 6 shows the EFs in plant ash at mined deposits of the North Caucasian geochemical province in relation to the regional abundance, illustrating the extent of uptake of elements by plants. Zn and Mo levels are increased in plants at all the deposits; Co is also accumulated at all deposits except for areas of Hg mineralization, and the concentration of Sr often increases. This can be explained by the combined effect of a number of factors:

1. Biogenic accumulation plays a significant role in all these elements.
2. The content of Mo, Co, and Zn in the province′s plants is reduced if comparing by EFs at the developed deposits.
3. The values of ionic radii and energy coefficients allow free migration over relatively long distances in an accessible form for plants.
4. The elements are also characterized by accumulation on sorption barriers, which is especially important for nonmetallic clay deposits. The mining of quarries for the extraction of nonmetallic raw materials increases the area of contact of vadose water with clays, forming new sorption barriers.
5. The values of EFs of the main ore elements for plants at the Buron, Urup, and Sakhalinskoe deposits are 1.5–4.0 times higher than in the corresponding fields (Table 1 and Table 6). This may also further indicate a single process that led to the formation of deposits and surrounding ore fields.

**Figure 6:**
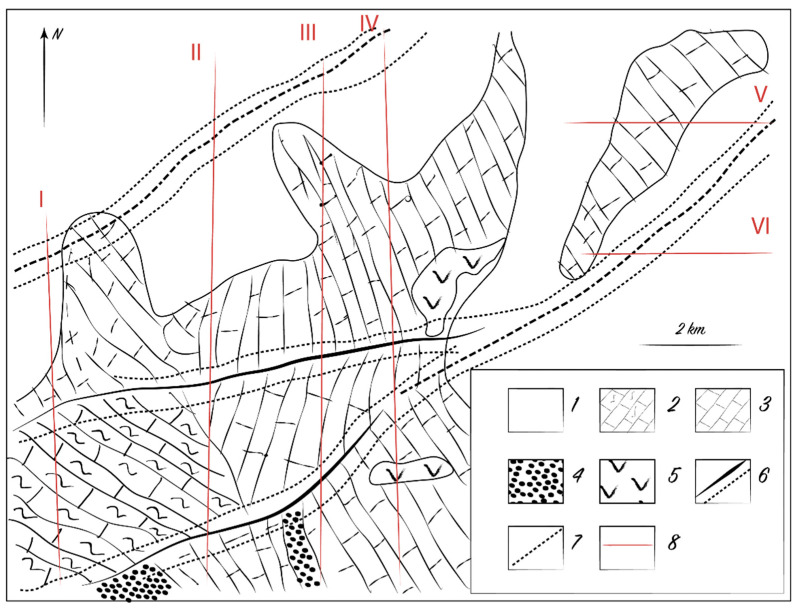
Biogeochemical anomalies over the disjunctive faults at the Telmanskoe ore field, Dzungarian Alatau. Symbols: 1—loose deposits, 20 m thick; 2—calcareous clayey shales; 3—limestones; 4—dolomites; 5—diorite porphyrites; 6—confirmed disjunctive faults; 7—presumable disjunctive faults; 8—biogeochemical Pb–Zn anomalies; 9—sampling transect lines and their numbers. The roman numerals (I–VI) show the transect numbers.

Tectonic disjunctive faults and schist-forming processes create peculiar geochemical stress zones for plants. As Figure 6 shows, linear biogeochemical anomalies developed as a response of plants to changing concentrations and modes of occurrence of chemical elements in fault zones with displacements from several to hundreds of meters. The zones of displacement and schist formation can be covered with unconsolidated sediments up to 100 m thick. Waterborne metals from the tectonized rocks migrate through cracks in such zones. Metals are subsequently deposited along the fault zones on evaporation barriers that become new stressors. In this case, the increased metal content and the changing modes of occurrence (water solutions instead of minerals) in the circulating flow are the most important factors affecting the geochemistry of plants. The content of indicator elements in plants is usually slightly higher than the local background and linear biogeochemical anomalies are detected by minimal anomalous contents (*C_an_ =*$\;\bar{x}$ *+ S*) in a large number of samples. When metal-rich rocks are displaced in ore regions, the most contrasting biogeochemical anomalies emerge. The contents of the main ore elements in the ash of plants affected by regional faults in such cases often exceed the anomalous concentrations for single samples. We especially note that even with a thick unconsolidated cover, biogeochemical anomalies are almost a vertical expression of faults. Consequently, the migration of solutions containing high concentrations of metals occurred vertically. The presence of faults detected by biogeochemical data in Southern Kazakhstan was confirmed by the results of drilling.

**Figure 7:**
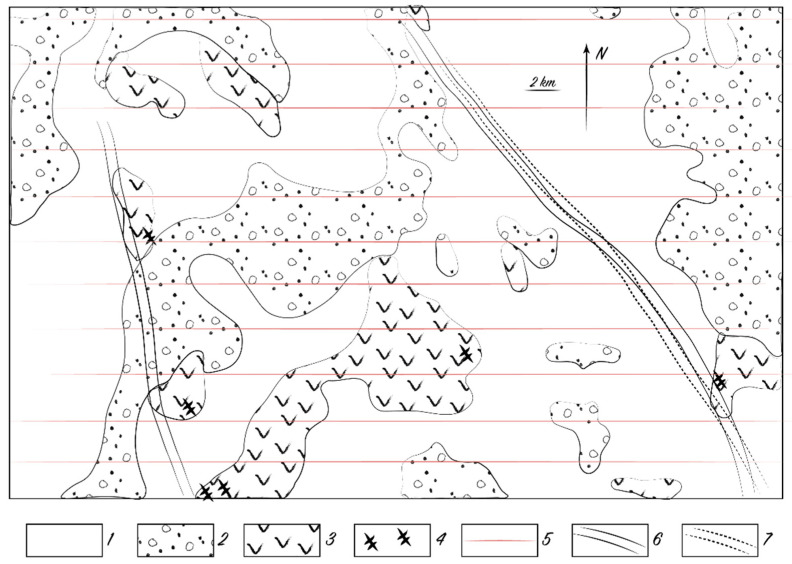
Linear biogeochemical anomalies over the schist-forming zones at the Alkamergenskoe ore field, Kazakh Uplands. Symbols: 1—loose deposits, 5 m thick; 2—Paleogene sandstones and gritstones; 3—effusive rocks; 4—schist-forming zones; 5—sampling transect lines; 6—Zn biogeochemical anomalies; 7—Mo biogeochemical anomalies.

Biogeochemical anomalies also occur over schist-forming zones (Figure 7). The content of chemical elements in these cases is usually abnormal for 2–9 correlating samples, and the elements themselves are among the most mobile and common in shale rocks.

**Figure 8:**
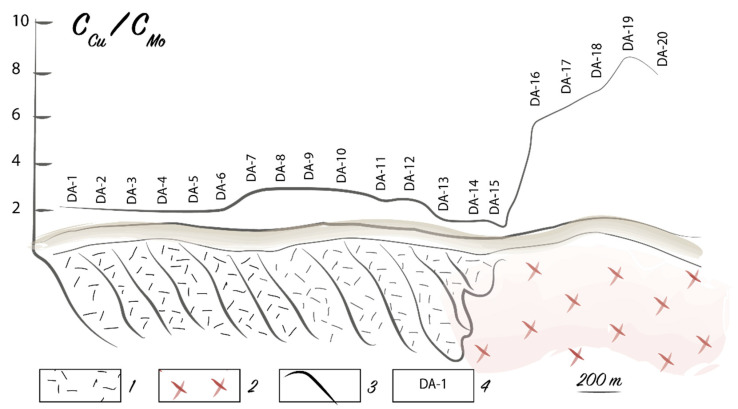
Proportion of Cu and Mo content in the ash of meadowsweet (*Spiraea hypericifolia* L.) over the changing bedrocks in the Dzungarian Alatau. Symbols: 1—effusive and tufogenic rocks; 2—granites; 3—proportion curve; 4—sample numbers.

It is commonly known that the composition of bedrock affects the content of chemical elements in plants. Rankama [59] suggested that changes in the Fe or Mn content of bedrock could be related to the change in Fe or Mn in plant ash; in addition, Warren et al. [60] proposed a change in the composition of the subsoil by changes in element concentrations of plant ash. Improvement in analysis has allowed the detection of almost all elements in plants over various rocks. A change in the geological and geochemical conditions of plant growth affects the laws of biological linkage between the elements (Figure 8). Therefore, a change in the bedrock over which the plants grow should cause a change in the ratio of metals in the ash of the plants.

Summarizing, we assert that the degree of exposure and thickness of a cover over the ore deposits, the content of indicator elements in the ores and primary halos in the ore-containing strata, the landscape and geochemical features of sites, types of tested plants and their biogeochemical features, and the behavior of chemical elements in the supergene zone are of prime importance for the element uptake in the soils over mineral deposits.

### 3.4. General Biogeochemical Accumulation Patterns

**Figure 9:**
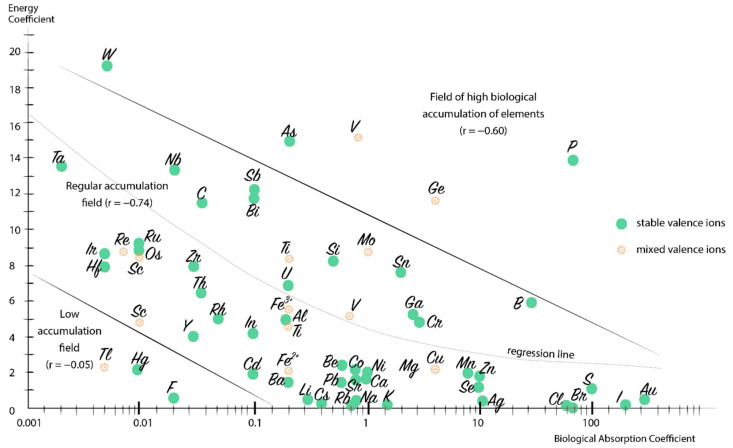
Accumulation of the chemical elements by plants as a function of the energy coefficient and the common logarithm of the biological absorption coefficient value.

We assume that the biological absorption coefficient (BAC) depends not only on the element content in soils and the characteristics of a particular plant species, but also on the values of the energy coefficients (Figure 9), i.e., on the values of ionic radii and valences [61]. The half-logarithm graph based on more than 10,000 analyzed soil–plant pairs reflects the balance: with an increase in the energy coefficients of the chemical elements, their BACs decrease sharply. Thus, we believe that a general relationship has been established between the chemical element accumulation by plants and the characteristics of the ions. With the primary role of ions in plant nutrition, we assume the presence of general laws of the chemical element migration, both in the mineral part of the biosphere and in living organisms that corresponds well to recent findings [62,63,64].

Analysis of the graph allows for identification of three fields: (i) regular biological accumulation (the vast majority of the chemical elements); (ii) high biological accumulation (P, As, V, and Ge); and (iii) low biological accumulation (Tl, Hg, and F). The correlation coefficient between the values of the energy coefficients and lg(BAC) of the elements in the field of normal accumulation is quite high, r = −0.74. The second and third accumulation fields can be associated both with the biological role of elements and with more global causes, up to the features of the origin of life on Earth. For that matter, further in-depth study of the features of the chemical element accumulation by living organisms at biogeochemical barriers is required, with a special emphasis on the structure of atoms and ions of these elements.

The ranked BACs of the elements in the North Caucasus geochemical province is Ag_5.4_–Mn_3.1_–Ba_1.4_–Cu_1.2_–Sr_1.2_–Zn_1.1_–Ni_0.9_–W_0.9_–Ge_0.5_–Mo_0.5_–Pb_0.5_–Cr_0.4_–Sn_0.4_–Li_0.3_–Co_0.2_–Ti_0.2_–V_0.1_. The BAC value greater than one indicates the accumulation of a specific element by a plant, and therefore the formation of a biogeochemical barrier. These data indicate that only 6 of the 17 elements considered are accumulated in the province′s plants at concentrations higher than in soils: Ag, Mn, Ba, Cu, Sr, and Zn. According to the Perelman’s classification [65], they are the elements of strong biological accumulation (Sr and Zn) and medium biological capture (Mn, Ba, and Cu). For elements with the highest EFs in the soils of the province (Pb, Cu, Zn, and Co), the values of BACs are minimal; moreover, Pb and Co concentrations in plants are lower in soils. According to the data obtained, it turns out that the elements that are in high concentrations in the soils of the province are absorbed by plants in smaller quantities than the average plants of the Earth. This is despite the fact that these elements migrate in the supergene zone mainly in the form of cations and the biogenic accumulation plays a significant role in their history (except for Pb).

Nonetheless, the regional abundance of Cu in the soils of the province is 2.6 times higher than the global abundance, and its local abundance in plants is 3 times less than the global level. Therefore, an increase or decrease in the content of elements in the growing environment does not always lead to similar changes in plants.

## 4. Conclusions

Biogeochemical studies of the North Caucasus, Dzungarian Alatau, Kazakh Uplands, and Karatau Mountains revealed features of the chemical element uptake by the local flora. Element accumulation patterns of native plants under the natural geochemical stress depend not only on the element content in soils and the characteristics of a particular species, but also on the values of ionic radii and valences; with an increase in the energy coefficients of the chemical elements, their accumulation by plants decreases sharply. In an oxidative environment and under elevated concentrations of highly and moderately mobile cationic aquatic migrants like Pb, Cu, and Zn in soils, with larger ionic radii and relatively low energy coefficients, can migrate over long distances predominantly as complex ions. Thus, internal factors contribute to the plant accumulation of these elements over vast areas. Considering phytoremediation prospects, we note that the ability of plants to uptake these elements from solutions permit soil remediation in large pollution zones. Application of hyperaccumulating species for mining site soil treatment depends on a number of external and internal factors that can strengthen or weaken the stressful situation, e.g., the exposure degree and thickness of loose deposits over the ores, the content of elements in the ores and primary halos in the ore-containing strata, the landscape and geochemical features of sites, and the chemical element migration patterns in the supergene zone.

## Figures and Tables

**Table 1:** Enrichment factors (EFs) of the chemical elements in the ash of woody plants from the ore fields of the North Caucasus geochemical province, calculated in relation to the regional abundances of the province.

Deposit	Series of EF Increase
*Buron*, lead–zinc	Mo_3.8_–Zn_2.8_–Pb_2.6_–Sn_2.5_–Li_2.4_–Co_2.3_–W_2.2_–Cu_1.6_–Sr_1.4_–Ge_1.0_–Ni_0.7_ *–Ba_0.5_–V_0.5_–Mn_0.5–_Ti_0.4_–Ag_0.3_–Cr_0.2_
*Urup*, copper–zinc	Ag_4.6_–W_2.2_–Sr_2.1_–Ba_1.8_–Zn_1.8_–Cu_1.7_–Co_1.4_–Li_1.4_–Ge_1.0_–Ni_1.0_–Sn_1.0_–Mo_0.8_–V_0.8_–Mn_0.7_–Pb_0.7_–Ti_0.4_–Cr_0.1_
*Sakhalinskoe*, mercury	Sr_2.2_–Mo_1.9_–Zn_1.8_–Ba_1.4_–Co_1.4_–V_1.3_–Ge_1.0_–Sn_0.8_–Ti_0.8_–Ag_0.7_–Ni_0.7_–Li_0.6_–Pb_0.5_–Mn_0.4_–Cr_0.2_

* Enrichment factors below unity are traditionally considered as a multiplicative inverse, however, depletion factors are above unity. The decimal numbers shown reflect the dispersion on a scale of the province.

**Table 2:** Biological absorption coefficients (BACs) of the chemical elements in the ash of woody plants from the ore fields of the North Caucasus geochemical province.

Deposit	Series of BAC Increase
*Buron*, lead–zinc	Mo_2.5_–Mn_2.0_–Sr_1.7_–W_1.7_–Cu_1.3_–Li_1.3_–Zn_1.2_–Ag_1.0_–Ni_1.0_–Ba_0.7_–Ge_0.7_–Co_0.5_–Sn_0.3_–Cr_0.2_–Pb_0.2_–Ti_0.1_–V_0.1_
*Urup*, copper–zinc	Ag_16.0_–Mn_3.8_–Ba_2.7_–Sr_2.3_–Zn_1.9_–Cu_1.7_–W_1.2_–Ni_0.8_–Mo_0.5_–Ge_0.5_–Co_0.4_–Li_0.4_–Pb_0.4_–Sn_0.4_–Cr_0.1_–Ti_0.1_–V_0.1_
*Sakhalinskoe*, mercury	Ag_5.0_–Cu_2.8_–Sr_2.6_–Zn_1.7_–Ba_1.6_–Mn_1.3_–Mo_1.0_–Ni_0.6_–Sn_0.4_–Pb_0.2_–Ti_0.1_–V_0.1_

**Table 3:** Metal content (%) in the ash of plants growing over rocks with varying Pb, Zn, and Cu levels in the Karatau Mountains.

Plants	Ore-Free Rocks			Low-Grade Ore			High-Grade Ore		
	Pb	Cu	Mo	Pb	Cu	Mo	Pb	Cu	Mo
Cherry (*Cerasus fruticosa* Pall.)	-	-	-	4.0 × 10^−3^	8.0 × 10^−3^	1.0 × 10^−4^	4.0 × 10^−2^	7.0 × 10^−3^	1.7 × 10^−4^
Meadowsweet (*Spiraea hypericifolia* L.)	1.3 × 10^−3^	1.4 × 10^−3^	5.0 × 10^−4^	6.9 × 10^−3^	7.4 × 10^−3^	1.0 × 10^−4^	2.5 × 10^−2^	6.2 × 10^−3^	1.0 × 10^−3^
Sophora (*Pseudosophora alopecuroides* (L.) Sweet)	1.1 × 10^−3^	2.3 × 10^−3^	1.5 × 10^−3^	6.0 × 10^−3^	6.4 × 10^−3^	-	1.3 × 10^−2^	6.4 × 10^−3^	-

**Table 4:** Lead content in the ash of cherry (*Cerasus fruticosa* Pall.) growing over polymetallic ore bodies with different mineral composition in the Karatau Mountains.

Site	Average Pb Content in the Bedrock, %	The Proportion of the Total Pb Content in the Rock by Containing Minerals, %				Average Pb Content in the Biogeochemical Anomaly, %
		Galena (PbS)	Plumbojarosite (PbFe_6_[SO_4_]_4_[OH]_12_)	Anglesite (PbSO_4_)	Cerussite (PbCO_3_)	
K1	3.90	33.0	8.8	18.0	38.0	5.3 × 10^−3^
K2	3.10	1.8	1.8	9.7	80.1	2.2 × 10^−2^
K3	0.14	-	70.0	10.0	20.0	8.6 × 10^−3^

**Table 5:** Metal content in the ash of saltwort (*Salsola arbuscula* Pall.) in changing geomorphological and geochemical conditions of Central Kazakhstan.

Landform	Element	Content %			
		Average	Anomalous		
			Number of Correlating Samples		Single Samples
			9	2	
Subhorizontal surface	Cr	0.002	0.003	0.005	0.006
Valley-shaped lowland		0.001	0.002	0.003	0.0036
Talus slope	Ni	0.0005	0.001	0.003	0.007
Valley-shaped lowland		0.0006	0.0009	0.0012	0.0015
Sheetwash slope	Cu	0.007	0.009	0.011	0.013
Subhorizontal surface		0.008	0.014	0.023	0.04

**Table 6:** Enrichment factors (EFs) of the chemical elements in the ash of leaves and needles of plants from the mined deposits of the North Caucasus geochemical province, calculated in relation to the regional abundances of the province.

Ag	Ba	Co	Cr	Cu	Ge	Li	Mn	Mo	Ni	Pb	Sn	Sr	Ti	V	W	Zn
*Urup* copper–zinc mine																
2.6	0.4	2.3	0.2	3.0	1.0	1.2	1.0	2.3	0.7	1.0	1.0	1.8	0.6	1.1	2.2	14.5
*Perevalnoe* mercury mine																
0.1	1.5	0.9	0.4	1.1	0.1	0.3	1.2	3.1	0.8	0.6	1.0	2.4	0.5	0.8	2.2	1.6
*Sakhalinskoe* mercury mine																
1.1	0.8	0.7	0.4	1.5	1.0	0.9	0.3	1.5	0.5	0.4	0.8	3.6	0.8	1.7	2.2	2.8
*Buron* lead–zinc mine																
0.9	0.6	9.1	1.0	1.7	1.0	2.4	2.0	3.1	1.0	4.1	3.0	1.2	2.5	4.4	2.2	7.9
*Gelendzhik* clay mine																
0.1	0.5	1.4	0.2	1.0	1.0	2.4	0.2	1.9	0.4	0.5	2.0	4.8	0.4	0.5	2.2	2.4
*Krymsk* clay mine																
0.4	0.6	4.3	0.4	0.8	1.0	3.6	1.6	3.8	1.1	0.6	2.0	0.8	2.0	2.5	2.2	2.4
*Kinzhal* clay mine																
0.1	0.2	11.4	0.1	0.9	1.0	3.0	0.3	1.5	1.4	0.4	1.5	16.6	0.2	0.5	2.2	4.0

## Data Availability

The data presented in this study are available in this article.
